# Supplementary material for: Strategies for the Management of Spike Protein-Related Pathology
Source: Microorganisms. 2023 May 17;11(5):1308. doi: 10.3390/microorganisms11051308 (PMC10222799; doi:10.3390/microorganisms11051308)
Supplement: Supplementary file 1 [file microorganisms-11-01308-s001.zip › microorganisms-2316768-supplementary.pdf]

Supplementary Table S1: Overview of Clinical Trials for long COVID-19 and COVID-19 vaccine injury.

Intervention class	Intervention	Clinical trials
Lifestyle	Exercise	NCT05566483<br>NCT05543408<br>NCT05530317<br>NCT05453175<br>NCT05360563<br>NCT05204511<br>NCT05094622<br>NCT04950725<br>NCT04900961<br>NCT04898205
	Mindfulness	NCT05566379<br>NCT05419219<br>NCT05268523<br>NCT05139979<br>NCT05107440<br>NCT04854772<br>NCT05119608<br>NCT05566379
	Wearable	NCT05507190<br>NCT05228665<br>NCT05019963
	Coaching	NCT05453201<br>NCT05422924<br>NCT05389592<br>NCT05338749<br>NCT05254301<br>NCT05167266<br>NCT04961333
	Physio/Rehab	NCT05453188<br>NCT05398692<br>NCT05373043<br>NCT05244044<br>NCT05238415<br>NCT05196529<br>NCT05040893<br>NCT05003271<br>NCT04996212<br>NCT04841759<br>NCT04649918<br>NCT05566483
	Transcranial direct current stimulation	NCT05389592<br>NCT05359770<br>NCT05289115<br>NCT05252481<br>NCT05126511<br>NCT05092516<br>NCT04890483

	Acupuncture	NCT05212688
	Vagus nerve stimulation	NCT05205577
	Electrical stimulation	NCT05200858<br>NCT05190718
	Homeopathy	NCT05104749
	Osteopathy	NCT05012826<br>NCT04928456*<br>NCT05069636*
	Singing	NCT04810065
Probiotics	Feacal Microbiota Transplant	NCT05556733
	Probiotic supplement	NCT05080244<br>NCT05195151*
Plasmapheresis	Plasma transfer	NCT05543590<br>NCT05445674
Anti-inflammatory /antioxidant /mitochondrial /metabolism modulators	Echinochrome A	NCT05531019
	Ibudilast	NCT05513560
	Mitoquinone	NCT05373043
	Prospekta	NCT05074888
	Coenzyme Q10	NCT04960215
	RSLV-132	NCT04944121
	Niagen	NCT04809974
	NAD+	NCT04604704
	metformin	NCT03996538*
	Ketogenic diet	NCT05163743*
Blood Thinners	Pentoxifylline	NCT05513560
	Atorvastatin (statin)	NCT04904536
	Plant stanol esters	NCT04844346

Immunomodulators	Temelimab	NCT05497089
	human immunoglobulin	NCT05220280
	MAbPACs	NCT05013723
	Sirolimus (rapamycin)	NCT04948203
	Ampion	NCT04880161
	Montelukast	NCT04695704
	Leronlimab	NCT04678830
	Zilucoplan	NCT04382755
	ergoferon	NCT05069649
	mycophenolate mofetil/mycophenolic acid or azathioprine	NCT05060991*
	Rituximab	NCT04877496*
	LYT-100 (Deupirfenidone)	NCT04652518
Heart medication	Ivabradine	NCT05481177
	Metoprolol Succinate	NCT05096884
Fibromyalgia medication	TNX-102 SL	NCT05472090
Hemp/CBD	Xltranplus, Xltran	NCT05467904
	CBD	NCT04997395<br>NCT04828668<br>NCT04777981
Oxygen therapies	portable oxygen concentrator	NCT05212831
	Hyperbaric oxygen	NCT04905888<br>NCT04842448
Stem cells	bone marrow-derived mesenchymal stem cells	NCT05116761
	Allogeneic Adipose-Derived Mesenchymal Stem Cells	NCT04992247
Antidepressant	Vortioxetine	NCT05047952

	Pimozide (For tinnitus specifically)	NCT05507372
Nasal spray	S-1226	NCT04949386
	Sodium pyruvate	NCT04871815
Adaptogens	ADAPT-232	NCT04795557
Flavonoids	Geneisten (BIO 300)	NCT04482595
	Mushroom mixture (Fomitopsis officinalis and Trametes versicolor)	NCT04951336*
Biologics	Zofin (derived from amniotic fluid)	NCT05228899
Dietary support	Iron	NCT04915820*
Antibiotics	Azithromycin	NCT04699097

Clinical trials are conducted for a long period unless otherwise stated. Clinical trials are for long Covid unless otherwise stated. *Vaccine immune response.
